# Supplementary material for: Leaf Carbohydrate Metabolism Variation Caused by Late Planting in Rapeseed (Brassica napus L.) at Reproductive Stage
Source: Plants (Basel). 2022 Jun 27;11(13):1696. doi: 10.3390/plants11131696 (PMC9268982; doi:10.3390/plants11131696)
Supplement: Supplementary file 1 [file plants-11-01696-s001.zip › plants-1759576-supplementary.pdf]

Supplementary Table S1. **Mean value of each physiological trait in 2019 and 2020.** The different lowercase letters in each column indicate significant difference at $P < 0.05$ according to Duncan's test.

Planting date	2019							2020						
	Days after anthesis													
	0	7	14	21	28	35	42	0	7	14	21	28	35	42
	Leaf dry matter (g $plant^{-1}$)													
15 September	7.11a	5.49b	4.77c	3.93ab	2.25bc	1.13b	0.61b	7.85a	7.49b	6.02c	3.50b	2.87b	2.05ab	0.62b
1 October	7.90a	8.78a	9.23a	5.17a	3.62a	2.31a	0.75a	8.57a	8.95a	10.71a	6.13a	5.76a	2.83a	0.68ab
15 October	5.78b	5.03b	6.88b	3.79b	2.73b	1.83ab	0.86a	5.14b	6.23c	7.67b	3.88b	3.41b	2.34a	0.83a
30 October	0.85c	2.11c	6.22bc	1.75c	1.55c	1.21ab	0.76a	2.85c	3.43d	7.05bc	1.78c	1.54c	1.07b	0.67ab
	Chlorophyll content (mg g^{-1} FW)													
15 September	1.16a	1.25a	1.23b	1.12b	0.89b	0.65c	0.58c	1.28a	1.33a	1.25b	1.26b	0.99b	0.72c	0.62c
1 October	1.16a	1.32a	1.44a	1.29a	0.85b	0.65c	0.56c	1.11b	1.32a	1.57a	1.49a	0.94b	0.59d	0.67c
15 October	0.93b	1.13b	1.22b	1.18ab	0.84b	0.83b	0.80b	0.82c	1.24a	1.21b	1.10b	0.98b	0.85b	0.81b
30 October	0.84b	0.75c	1.13c	1.13b	1.07a	0.94a	1.02a	0.83c	0.85b	1.24b	1.13b	1.27a	1.09a	1.01a

Total soluble sugar content (mg g^{-1} DW)														
15 September	28.1a	16.2a	12.1c	12.4b	9.2d	8.3d	5.1d	30.0a	16.5a	13.3c	14.4c	9.3d	11.4c	9.5c
1 October	27.8a	15.9a	13.9b	16.0a	13.2c	10.7c	6.9c	29.5a	19.2a	18.7a	15.0bc	13.6c	12.6c	10.6c
15 October	13.6b	14.9a	15.7a	17.1a	14.6b	13.8b	12.8b	13.0b	10.7b	16.1b	18.1a	16.9b	17.2b	14.5b
30 October	9.9c	8.3b	15.1ab	15.8a	16.5a	17.4a	15.5a	8.3c	7.4c	11.1c	16.5ab	18.5a	19.5a	20.2a
Acid invertase activity (nmol h^{-1} mg^{-1} protein)														
15 September	302.4b	185.9b	89.0a	57.8b	75.6ab	58.4a	62.1ab	218.6c	201.9b	87.9a	44.8b	32.9b	41.7c	67.4a
1 October	389.8a	223.1a	97.3a	90.1a	63.5b	45.6b	69.3a	338.5a	246.5a	89.9a	39.2b	62.7a	29.4d	71.4a
15 October	246.0c	184.6b	50.2b	61.1b	31.9c	44.4b	45.5c	263.5b	145.4c	29.0c	43.4b	38.1b	102.8a	48.2b
30 October	234.9c	126.3c	56.1b	55.8b	78.8a	59.5a	49.2bc	217.2c	94.4d	54.9b	58.2a	57.9a	63.1b	41.8c
Neutral invertase activity (nmol h^{-1} mg^{-1} protein)														
15 September	57.6b	48.9c	108.9a	54.2a	49.8a	54.9a	104.4a	34.9c	74.5b	101.4a	50.2b	41.7c	57.6a	118.8a
1 October	58.0b	97.7a	91.8b	42.7b	40.3b	48.8ab	81.0b	53.9b	96.2a	92.7ab	58.5a	68.7a	48.5b	96.6b
15 October	75.6a	91.8a	83.9b	58.7a	473a	44.1b	64.5c	50.6b	78.9b	88.9b	46.7b	55.1b	20.0c	70.4c
30 October	78.6a	63.7b	44.2c	39.8b	27.9c	30.8c	57.1c	72.5a	47.8c	72.5c	48.5b	23.9d	23.8c	65.1c

	Sucrose phosphate synthase activity (nmol h^{-1} mg^{-1} protein)													
15 September	239.4a	125.1b	127.2c	143.6b	145.7b	121.3b	100.3c	133.1b	215.0a	128.9b	106.6c	130.1ab	128.0b	74.1c
1 October	138.4c	184.6a	169.2a	128.2b	129.8c	113.1b	81.4d	97.6c	97.1c	154.3a	125.6b	129.7b	104.5c	80.3c
15 October	111.7d	105.8b	147.7b	167.9a	163.6a	141.8a	127.4a	121.0b	123.3b	132.3b	149.4a	163.3a	156.2a	121.8a
30 October	215.8b	190.6a	112.1c	75.2c	75.3d	107.0b	115.4b	175.0a	104.3c	99.2c	68.9d	48.9c	54.8d	97.8b
	Sucrose synthase activity (nmol h^{-1} mg^{-1} protein)													
15 September	345.8b	249.2b	168.0d	262.2d	296.4d	387.1c	274.2a	164.0c	202.2c	166.0d	204.5c	320.3d	363.1a	291.9a
1 October	293.7c	225.0c	247.2c	283.8c	321.5c	468.0a	194.4c	220.9b	253.3b	332.8a	273.3b	484.6a	262.0c	228.1b
15 October	249.0d	216.5c	300.9a	331.0a	407.3a	334.3d	245.6b	307.3a	195.4c	274.4b	329.8a	360.4c	274.0c	251.1ab
30 October	405.1a	361.5a	277.3b	309.0b	357.3b	437.7b	291.9a	288.8a	329.2a	248.9c	298.6ab	386.1b	329.7b	272.2a

Starch phosphorylase activity (nmol h^{-1} mg^{-1} protein)														
15 September	47.8a	19.8d	81.8a	79.2b	90.8b	14.8a	9.1bc	56.5a	26.6c	39.7a	135.8b	146.4c	10.6c	10.0ab
1 October	42.4b	44.1a	44.4c	102.5a	149.4ab	13.2ab	11.4a	32.2c	23.6c	25.4c	95.3c	182.9b	19.7a	11.4a
15 October	12.4c	33.1b	24.6d	98.1a	231.0a	10.8c	10.3ab	46.3b	40.7b	26.9c	150.6a	223.1a	15.1b	8.2b
30 October	46.3a	28.3c	52.1b	41.6c	96.1b	11.5bc	8.0c	18.7d	80.8a	31.3b	94.5c	84.8d	12.7bc	10.5ab
ADP-glucose pyrophosphorylase activity (nmol h^{-1} mg^{-1} protein)														
15 September	122.8a	135.6b	94.9a	74.4c	53.6d	53.6c	63.6d	121.1a	158.2b	119.4a	85.7b	51.2d	54.2d	50.8d
1 October	118.0a	160.6a	91.5ab	82.5bc	61.8c	57.7c	67.8c	103.2b	181.7a	85.0c	88.6b	68.4c	63.8c	58.9c
15 October	49.6b	80.9c	87.7b	97.3a	135.4a	111.9a	113.9a	38.0c	65.8c	90.0b	101.9a	125.1a	105.8a	103.3a
30 October	32.6c	46.9d	76.2c	84.4b	116.0b	104.5b	84.8b	37.8c	57.1d	71.8d	87.9b	107.8b	97.7b	96.4b

.

Supplementary Figure S1

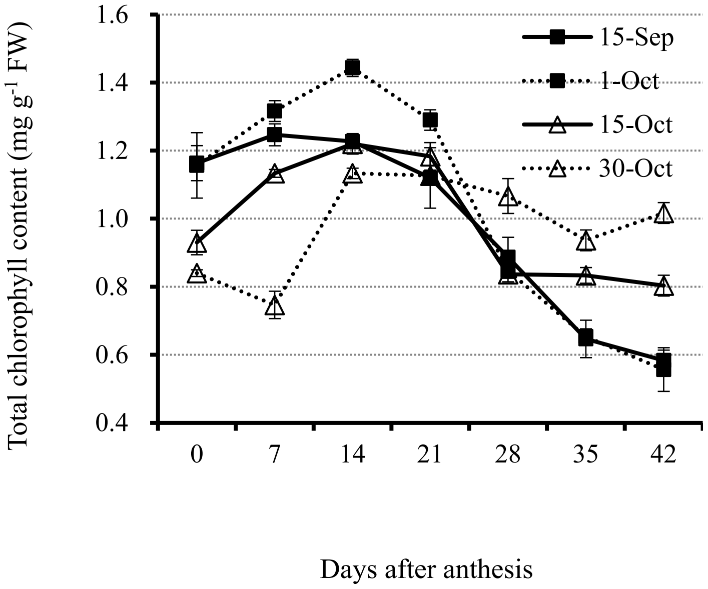

Leaf total chlorophyll content from anthesis with a 7 d interval under four planting dates—early, optimal, late, and very late (15 September, 1 October, 15 October, and 30 October)—in 2019. Bars of each value are standard error.

Supplementary Figure S2

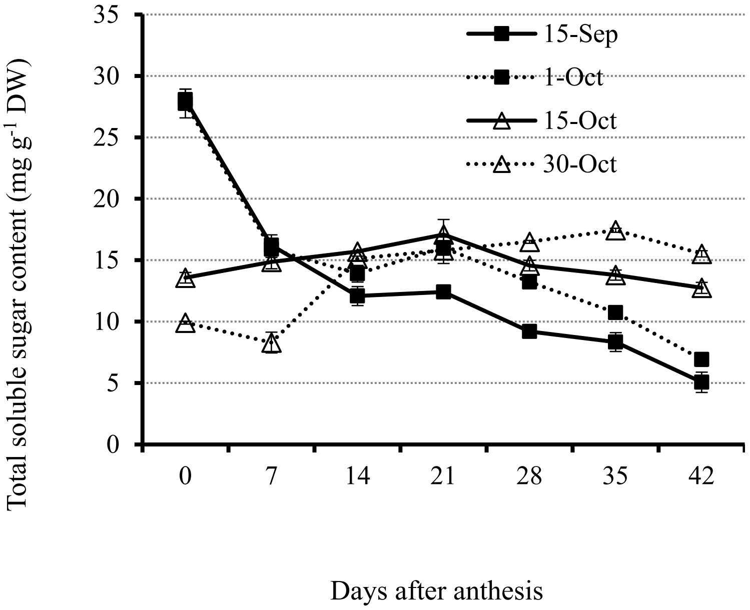

Leaf total soluble sugar content from anthesis with a 7 d interval under four planting dates—early, optimal, late, and very late (15 September, 1 October, 15 October, and 30 October)—in 2019. Bars of each value are standard error.

Supplementary Figure S3

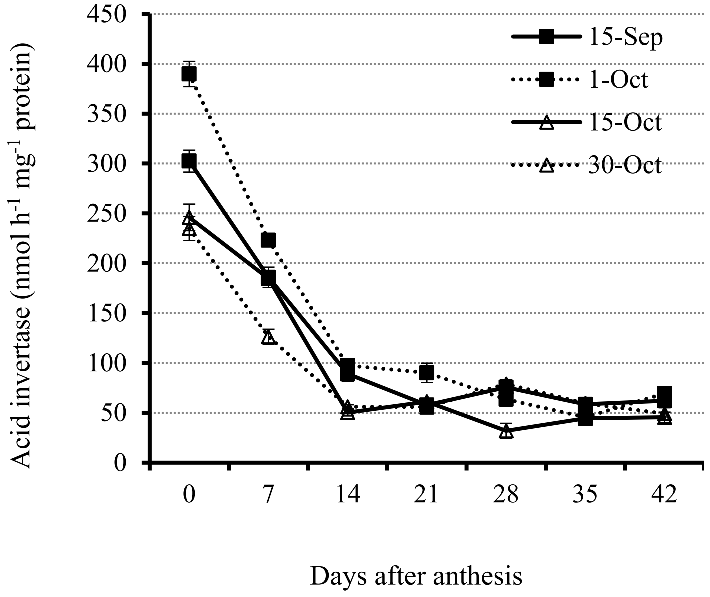

Leaf acid invertase activity from anthesis with a 7 d interval under four planting dates—early, optimal, late, and very late (15 September, 1 October, 15 October, and 30 October)—in 2019. Bars of each value are standard error.

Supplementary Figure S4

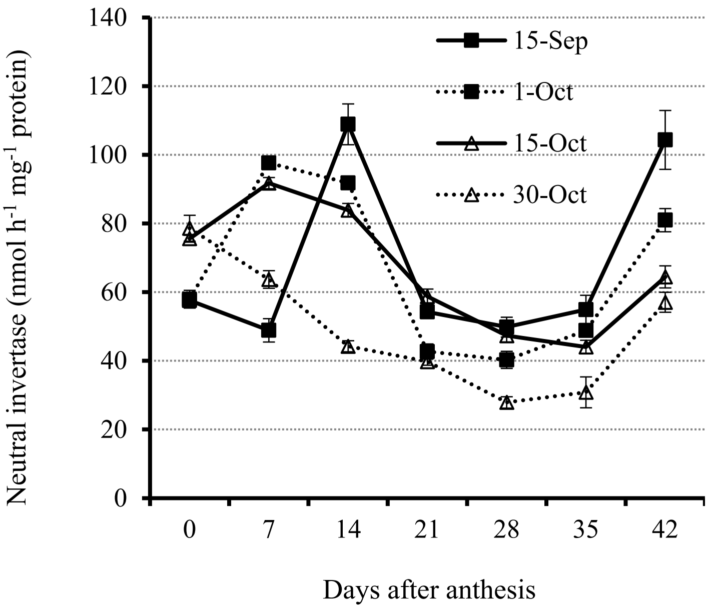

Leaf neutral invertase activity from anthesis with a 7 d interval under four planting dates—early, optimal, late, and very late (15 September, 1 October, 15 October, and 30 October)—in 2019. Bars of each value are standard error.

Supplementary Figure S5

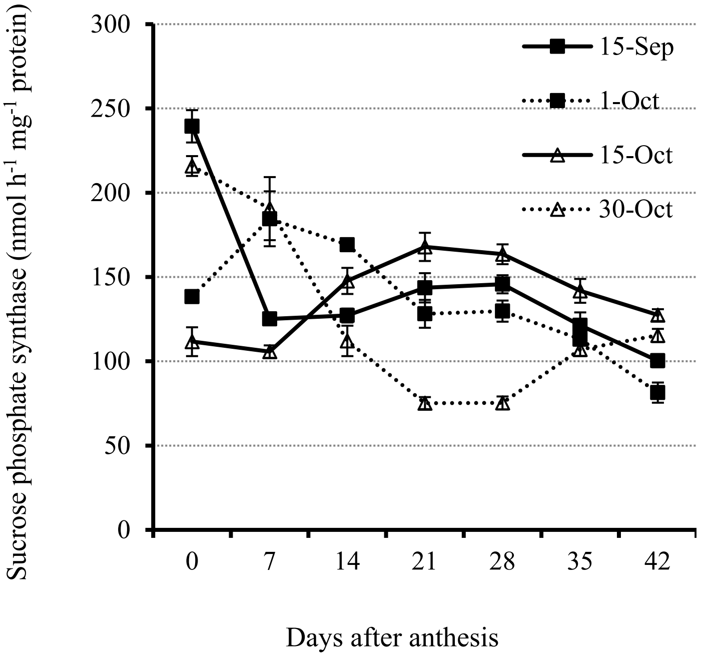

Leaf sucrose phosphate synthase activity from anthesis with a 7 d interval under four planting dates—early, optimal, late, and very late (15 September, 1 October, 15 October, and 30 October)—in 2019. Bars of each value are standard error.

Supplementary Figure S6

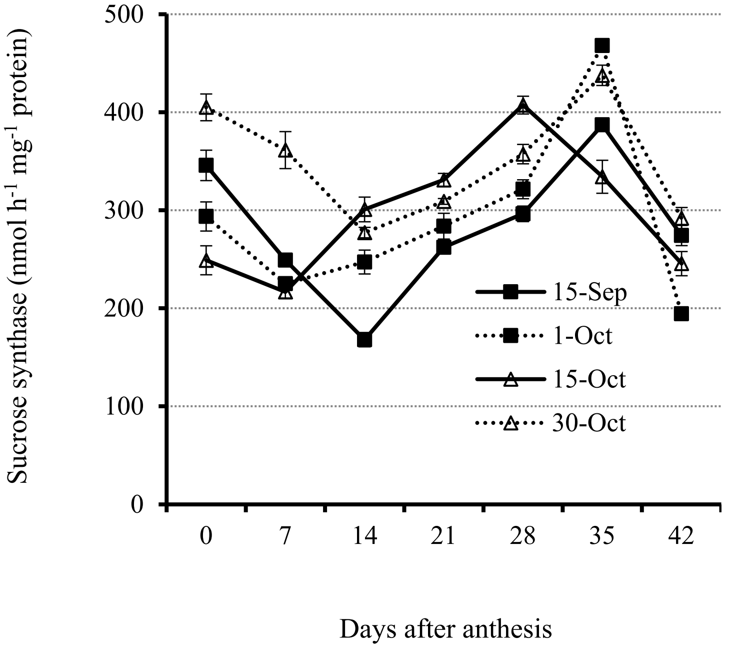

Leaf sucrose synthase activity from anthesis with a 7 d interval under four planting dates—early, optimal, late, and very late (15 September, 1 October, 15 October, and 30 October)—in 2019. Bars of each value are standard error.

Supplementary Figure S7

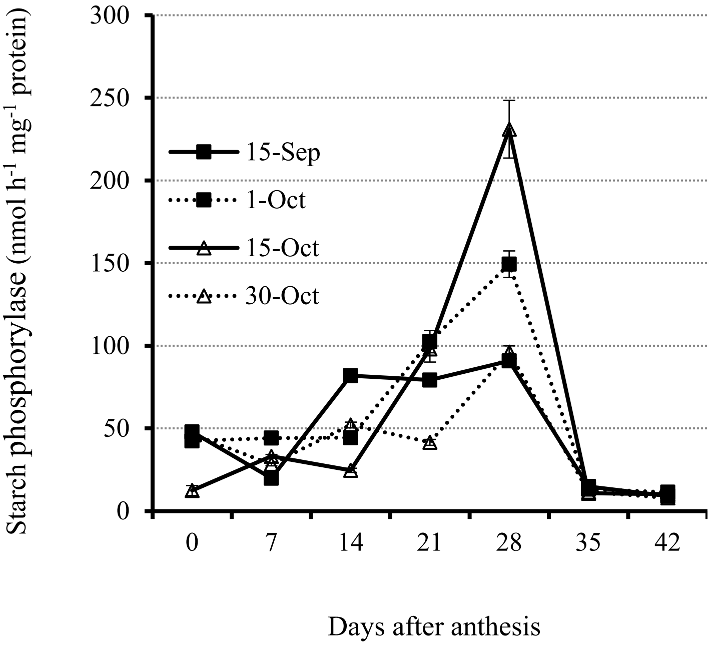

Leaf starch phosphorylase activity from anthesis with a 7 d interval under four planting dates—early, optimal, late, and very late (15 September, 1 October, 15 October, and 30 October)—in 2019. Bars of each value are standard error.

Supplementary Figure S8

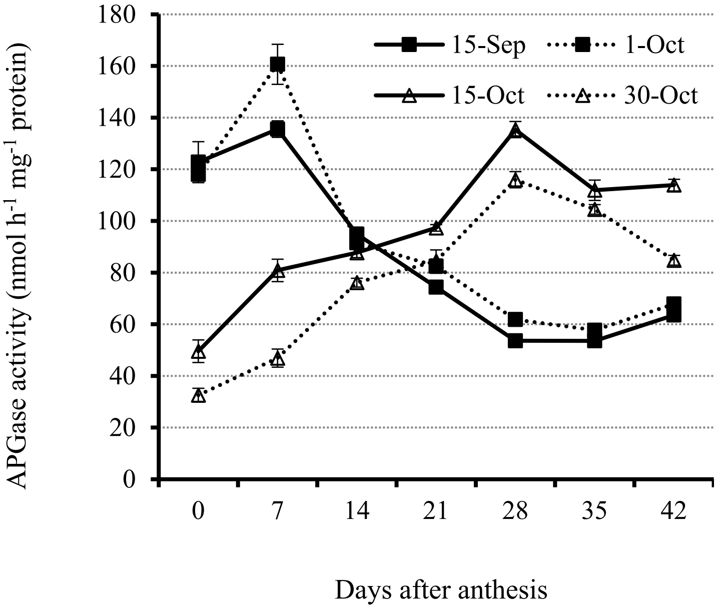

Leaf ADP-glucose pyrophosphorylase activity from anthesis with a 7 d interval under four planting dates—early, optimal, late, and very late (15 September, 1 October, 15 October, and 30 October)—in 2019. Bars of each value are standard error.
